# Genotyping of infectious bronchitis virus in Canada

**DOI:** 10.1177/10406387241265955

**Published:** 2024-08-06

**Authors:** Davor Ojkic, Leonardo Susta, Emily Martin

**Affiliations:** Animal Health Laboratory, University of Guelph, Guelph, Ontario, Canada; Department of Pathobiology, University of Guelph, Guelph, Ontario, Canada; Animal Health Laboratory, University of Guelph, Guelph, Ontario, Canada

**Keywords:** infectious bronchitis virus, genotyping, spike protein gene, variants

## Abstract

From 2014–2023, infectious bronchitis virus (IBV) was detected in 6,589 samples from Canada, and partial nucleotide (nt) sequences of the IBV spike protein (*S*) gene were determined for 1,678 samples. Based on their *S* gene nt sequence identities and origin, Canadian IBVs could be classified into 4 groups: 1) 50.3% were variant viruses related to strains described in the United States; 2) 45.6% were vaccine-like viruses; 3) 2.1% were Eurasian viruses; 4) 2.0% were Canadian variants. Outbreaks with IBVs related to strains CAL1734/04, 4/91, and DMV/1639/11 were often associated with more severe disease in all chicken commodity groups. With the emergence of numerous IBV strains, the severity of infection and number of affected flocks increased. Outbreaks with various IBV strains overlapped in their emergence, peaked, and regressed, but the introduction of DMV/1639/11 has resulted in a continuous field challenge since its first detection in 2015.

In Canada, disease associated with infectious bronchitis virus infection (IBV; *Coronaviridae*, *Gammacoronavirus*, *Gammacoronavirus galli*) was, for many years, limited to sporadic outbreaks. Between 2000–2011, IBV-positive samples that were genotyped at the Animal Health Laboratory (AHL; University of Guelph, Guelph, Ontario, Canada) were largely strains related to variant IBVs circulating in the United States at that time. However, in early 2012 and in 2013, an increased number of submissions related to IBV infection were reported.^13^ These cases involved various commodity groups and resulted in increased mortality due to respiratory disease and nephropathogenic involvement in broilers, and egg-production issues in egg-laying birds. The 2012–2013 outbreak was caused by the incursion of IBV strain 4/91, which became endemic in Ontario and reached a peak in 2013.^14^ The IBV 4/91 strain was first detected in Great Britain in 1990,^7^ and was later found in Asia and South America,^3^ but had not been identified previously in Canada. The field situation shifted when IBV re-emerged and, in 2016, IBV DMV/1639/11–like strains had become the most important viral chicken pathogen in Ontario causing severe disease affecting all chicken commodity groups. The outbreak caused by IBV DMV/1639/11–like strains was first detected in Ontario in a 23-wk-old layer flock experiencing production problems and increased mortality, then spread rapidly through Ontario^16^ and later involved other Canadian provinces.^9^

The IBV DMV/1639/11 strain had first been detected in 2011 in Delmarva broiler flocks^6^ and is believed to be descendent of IBV variants such as PA/Wolg/98 and PA/171/99, which caused nephropathogenic outbreaks in Pennsylvania in 1997–2000.^18^ However, IBV DMV/1639/11 infection is not only nephropathogenic but can cause severe respiratory disease, egg production issues, and has become the predominant variant in the United States.^12^ In addition, early infection with IBV DMV/1639/11 has been associated with “false layer syndrome”^15^ in female chickens, as well as testicular atrophy, epididymitis, and orchitis in male broiler–breeders.^5^ Here we describe and analyze the historical data related to IBV testing at the AHL in 2014–2023.

## Materials and methods

### Samples

From January 1, 2014–December 31, 2023, 10,217 samples were submitted to the AHL for IBV PCR testing. We focused on analyzing data from chicken samples submitted for diagnostic investigation purposes. Consequently, we excluded 148 samples from research projects and 48 samples from other species and analyzed data related to 10,021 diagnostic samples.

### Nucleic acid extraction and PCR

We extracted total nucleic acids (MagMAX-96 viral RNA isolation kit, MagMAX Express-96 magnetic particle processor; Applied Biosystems) with armored RNA enterovirus (Asuragen) as an internal control and used a real-time PCR as a screening test for IBV detection (AgPath-ID One-Step RT-PCR kit; Applied Biosystems) as described previously.^1^ We carried out PCR amplification and detection in a Light Cycler 480 (Roche). Samples with cycle thresholds (Cts) < 37 were considered positive, and samples with Cts ≥ 37 were considered inconclusive. If no amplification was detected (e.g., no Ct value was produced), the sample was reported as negative.

### Genotyping

We designed primers IBV_S_F-uni1_161125 (5′-GGTTGGCATYTACAHGGR-3′) and IBV_S_R-uni1_161125 (5′-TCTTGTRCRGTACCATTA-3′) with PrimerQuest Tool (Integrated DNA Technologies) using sequences of IBV strains that circulate in Canada. These primers amplified a 542-nucleotide (nt) fragment of the IBV spike protein (*S*) gene (nt 115-656 based on the *S* gene sequence from IBV 4/91; GenBank JN192154), encompassing *S* gene hypervariable regions 1 and 2. PCR amplification was carried out (One-step RT-PCR kit, Qiagen; Biometra T3 thermocycler, Analytik Jena), PCR products were visualized (E-Gel Imager; Thermo Fisher), and nt sequences of amplicons were determined at Laboratory Services Division, University of Guelph (Guelph, Ontario). We assembled and compared sequences with the SeqMan Pro and MegAlignPro modules of Lasergene (v.17.5.0; DNAstar). Sequences with ≥87% nt sequence identities clustered with proposed IBV lineages; sequences with <87% nt sequence identity to previously described IBV strains were considered unique variants.^17^

## Results

**Figure 1.**
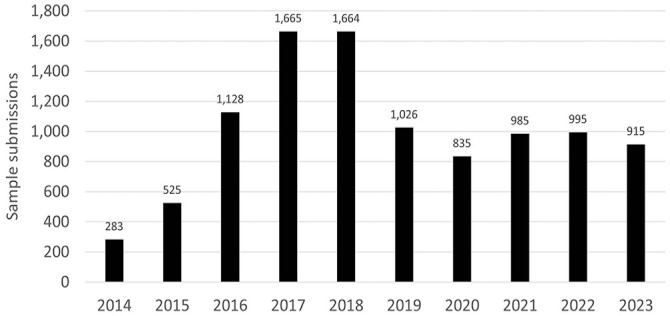
Frequency of sample submissions for infectious bronchitis virus PCR, 2014–2023.

Sample submissions for IBV PCR were at the lowest number in 2014 when only 283 samples were submitted; the highest number of samples (1,665) were tested in 2017 (Fig. 1). A large majority of samples (8,671 of 10,021; 86.5%) were from Ontario. The remaining 1,350 of 10,021 (13.5%) samples were from 8 Canadian provinces: Alberta, British Columbia, Manitoba, Newfoundland and Labrador, Nova Scotia, Quebec, and Saskatchewan. On the sample level, 3,432 of 10,021 (34.2%) samples were PCR-negative and 6,589 (65.8% ) were non-negative (5,445 were positive and 1,144 were inconclusive). The percentage of non-negative samples varied from the low of 115 of 283 (40.6%) in 2014 to the high of 1,214 of 1,664 (73.0%) in 2018.

**Figure 2.**
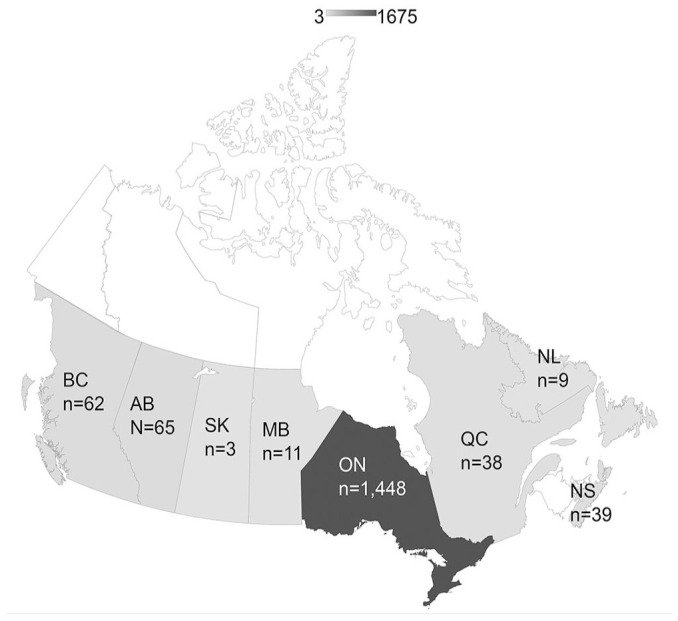
The origin of genotyped infectious bronchitis virus samples by Canadian province (AB = Alberta; BC = British Columbia; MB = Manitoba; NL = Newfoundland and Labrador; NS = Nova Scotia; ON = Ontario; QC = Quebec; SK = Saskatchewan (geographic origin was not known for 3 samples).

We genotyped 1,678 IBV-positive samples from 8 Canadian provinces (Fig. 2): 1,601 of 1,656 (96.7%) from commercial poultry and 55 of 1,656 (3.3%) from backyard flocks. Commodity group was not known for 22 samples. We submitted the nt generated sequences to GenBank (OR634981–OR635007, OR638360–OR639833, PP208634–PP208810). Based on their partial *S* gene nt sequences, 1,678 genotyped samples could be classified into 4 geographically and clinically demarcated groups belonging to 8 IBV lineages (Table 1): 1) 844 of 1,678 (50.3%) were variant IBVs related to strains described in the United States (DMV/1639/11, PA/Wolg98, CAL/1737-04, GA/08, CU/82792, GA/98, CA/K19-01179); 2) 766 of 1,678 (45.6%) were vaccine-like viruses (Massachusetts, Connecticut, Arkansas); and 3) 35 of 1,678 (2.1%) were Eurasian origin viruses (4/91); 4) 33 of 1,678 (2.0%) were Canadian/unique variants (QU/mv, BC/AHL14-023482, ON/AHL16-046445) that have not been reported elsewhere.

**Table 1.** Summary of infectious bronchitis virus genotyping results for 1,678 samples, 2014–2023.

Group/Strain	Lineage	2014	2015	2016	2017	2018	2019	2020	2021	2022	2023	Total
Vaccine-like												
Connecticut	GI-1	1	14	7	19	49	49	37	45	20	27	268
Massachusetts	GI-1	12	30	41	77	68	44	34	56	83	51	496
Arkansas	GI-9								1		1	2
U.S. variant												
DMV/1639/11	GI-17		1	31	127	113	101	43	61	61	81	619
PA/Wolg/98	GI-17		1	3								4
CAL/1737-04	GI-25	2	10	29	28	18	9	9	23	27	6	161
GA/08	GI-27							1				1
CU/82792	GIV-1			1	4	3	8	7	15	8	10	56
GA/98	GIV-1							1				1
CA/K19-01179	Unique variant				2							2
Eurasian												
4/91	GI-13	8	5	13	8			1				35
Canadian variant												
QU/mv	GI-20			5	4		1					10
BC/AHL14-023482	Unique variant	4	2		5				1	1	1	14
ON/AHL16-046445	Unique variant			5								5
Various	Unique variant		1		3							4
Total		27	64	135	277	251	212	133	202	200	177	1,678

**Figure 3.**
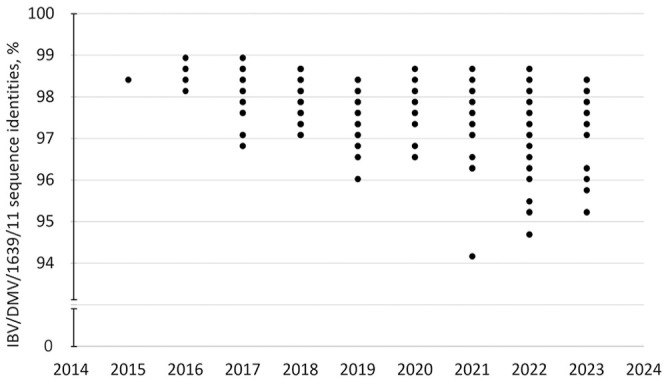
The drift of nucleotide sequence identities of infectious bronchitis virus (IBV) DMV/1639/11 from 2015–2023.

In commercial poultry, the highest proportion of IBV strains were U.S. variant strains (807 of 1,601; 50.4%) and vaccine-like viruses (755 of 1,601; 47.2%). This differed from backyard flocks in which the U.S. variant strains were well represented at 25 of 55 (45.5%), but vaccine viruses were detected at only 4 of 55 (7.3%; Table 2). The S1 sequence of the most frequently detected variant virus, DMV/1639/11, has been drifting since its introduction in both commercial and backyard flocks (Fig. 3).

**Table 2.** Distribution of infectious bronchitis virus genotyping results in backyard flocks and commercial poultry (commodity was not known for 22 samples).

Group/Strain	Lineage	Commercial poultry		Backyard flocks	
		*n*	%	*n*	%
Vaccine-like					
Conn	GI-1	266	16.6		0.0
Mass	GI-1	487	30.4	4	7.3
Ark	GI-9	2	0.1		0.0
U.S. variant					
DMV/1639/11	GI-17	603	37.7	5	9.1
PA/Wolg98	GI-17	4	0.2		0.0
CAL/1737-04	GI-25	143	8.9	17	30.9
GA/08	GI-27	1	0.1		0.0
CU/82792	GIV-1	55	3.4	1	1.8
GA/98	GIV-1	1	0.1		0.0
CA/K19-01179	Unique variant	0	0.0	2	3.6
Eurasian					
4/91	GI-13	26	1.6	8	14.5
Canadian variant					
QU/mv	GI-20	1	0.1	9	16.4
BC/AHL14-023482	Unique variant	12	0.7		0.0
ON/AHL16-046445	Unique variant	0	0.0	5	9.1
Various	Unique variant	0	0.0	4	7.3
Total		1,601		55	

## Discussion

Outbreaks associated with IBV infection have been occurring continuously in Ontario and other Canadian provinces since the early 2000s, but these outbreaks were rather sporadic, and their impact was limited.^8,14^ The introduction of IBV strains related to DMV/1639/11 in 2015 has resulted in a marked increase in the frequency of sample submissions for IBV testing reflecting the severe losses in all chicken commodities. It appeared that IBV DMV/1639/11 challenge was at a peak in 2017 when 127 genotyped samples from commercial birds were genotyped as IBV DMV/1639/11. In later years, the frequency of DMV/1639/11-genotyped samples remained high and is still associated with a large number of diagnostic submissions. Along with DMV/1639/11, an increased frequency of detection of CAL/1737-04 variants has occurred. It has been reported that in experimental settings, Mass/Conn vaccination can provide up to 80% protection against CAL/1737-04 challenge,^11^ but it appeared that, in field conditions in Canada, the level of protection is much lower, possibly as a result of further antigenic drift of CAL/1737-04 variants in Canada.

Not surprisingly, vaccine-like viruses were commonly detected in commercial birds in which IBV vaccination is used. On the other hand, vaccine-like IBVs were an infrequent finding in backyard flocks, in which vaccination is uncommon. Although the number of genotyped samples from backyard flocks was small, it was interesting to observe that the frequency of Canadian unique variants was high, at 18 of 55 (32.7%), compared to 13 of 1,601 (0.8%) in commercial flocks. Wild birds can harbor IBV-related viruses,^4^ but based on our results it is not clear if Canadian unique variant IBVs in backyard flocks could have originated from wild-bird coronaviruses or were introduced from commercial birds and evolved independently.

Egg-laying commercial flocks in Canada are hyperimmunized with repeated applications of live-attenuated viral vaccines followed by a booster with inactivated viral vaccines. Broilers are typically spray-vaccinated with live-attenuated products on day 1 in the hatchery, but field vaccination is done infrequently at the discretion of attending veterinarians. Only Massachusetts and Connecticut-derived IBV strains are available as live-attenuated viral vaccines in Canada. Inactivated vaccine formulations used as boosters are also available with the Arkansas IBV component. It appeared that these vaccines provided some, albeit low, levels of cross-protection against certain IBV variants (4/91, CAL/1734/04),^14^ possibly as a result of cross-reactivity involving cytotoxic T-lymphocytes.^2^ In clinical cases, the detection of various IBV strains overlapped in their emergence, peaked, and regressed over several years. However, DMV/1639/11-related outbreaks were often associated with more severe disease in all chicken commodity groups, and the number of affected flocks rapidly increased.

Sample collection was passive, but assumedly corresponded to the field situation. Given the size of the poultry industry in Ontario and the geographic proximity of our laboratory, most samples were from Ontario poultry producers. Still, 227 genotyped samples were from other Canadian provinces providing information about circulation of IBV strains throughout Canada. Based on its *S* gene nt sequence, the first IBV DMV/1639/11 virus that was detected in Canada in 2015 was 97% identical to its prototype counterpart. However, since being introduced, DMV/1639/11 strains have been continuously mutating, and the challenge could not be successfully mitigated by vaccines available in Canada.^10^ Consequently, alternative vaccination protocols will need to be contemplated should the field situation remain inadequately controlled.
